# Synovial hemangiohamartoma presenting as knee pain, swelling and a soft tissue mass: a case report

**DOI:** 10.1186/1752-1947-6-207

**Published:** 2012-07-18

**Authors:** Serkan Senol, Hakan Cift, Korhan Ozkan, Esat Uygur, Maria Silvia Spinelli

**Affiliations:** 1 Sb. Medeniyet University Goztepe Education and Research Hospital, Department of Pathology, Dr. Erkin street, Istanbul 34722 Turkey; 2 Department of Orthopaedic and Traumatology, Sb. Medeniyet University Goztepe Education and Research Hospital, Dr. Erkin street, Istanbul 34722 Turkey; 3 Department of Orthopedics and Traumatology, Catholic University, Via Eugenio Tansi, 67, 00135 Rome, Italy

**Keywords:** Synovial hemangiohamartoma, Knee, Hemorrhagic synovitis, Recurrent spontaneous hemarthrosis

## Abstract

**Introduction:**

We present a case of a patient with juxtaarticular hemangiohamartoma with a synovial extension associated with hemorrhagic synovitis and recurrent spontaneous hemarthrosis.

**Case presentation:**

A 21-year-old Caucasian woman was admitted to our hospital complaining of pain and swelling at her knee for 6 months. In the magnetic resonance imaging, T2-weighted and fat-suppressed scans revealed a mass with high signal intensity just posterior to the patellar tendon. We performed an excisional biopsy of the mass through an anterior longitudinal incision. Excised material included arterial and venous vascular structures, which were found to be spread among the fat, connective and peripheral nerve tissues microscopically.

**Conclusion:**

Although hemangiohamartomas are not true neoplasms, they may cause knee pain, swelling and hemarthrosis that warrant surgical resection. This lesion, although rare, should be considered in the differential diagnosis, especially in teenagers and young adults.

## Introduction

Synovial and subsynovial hemangiomas are very rare benign lesions that are classified by pathologists as hemangiohamartomas when admixed with fat, connective tissue and peripheral nerve structures [1,2]. The predominant type of vascular channel during pathological examination reveals the lesion as cavernous, capillary, arteriovenous or venous.

Recurrent non-traumatic hemarthrosis with pain and palpable mass are among the common findings experienced by the patient [3]. Magnetic resonance imaging (MRI) is usually the diagnostic tool for detection of the extent and characteristics of the lesion [4,5].

Complete open excision decreases the potential for local recurrence, but localized lesions can be treated arthroscopically [6-8]. In this article, we present a case of juxtaarticular hemangiohamartoma with a synovial extension associated with hemorrhagic synovitis and recurrent spontaneous hemarthrosis.

## Case presentation

A 21-year-old Caucasian woman was admitted to our hospital complaining of pain and swelling at her knee for the past 6 months. Her pain and swelling were especially aggravated by physical activities.

**Figure 1:**
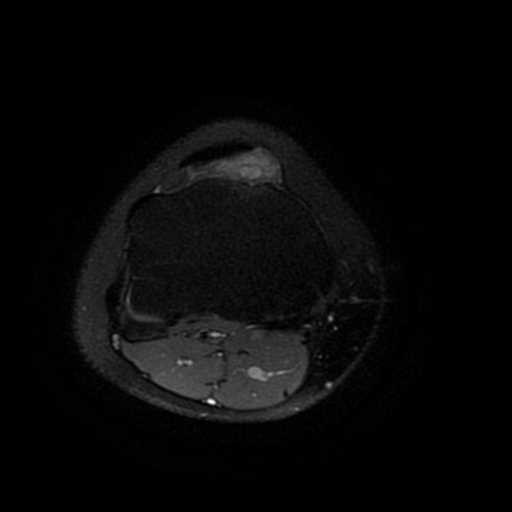
T2-weighted axial view of the knee displaying the lesion with a bright signal under the patellar tendon.

Her physical examination revealed that she had mild quadriceps atrophy and pain during terminal flexion of her knee, with mild swelling just beneath the patellar tendon. Her conventional radiographies displayed no abnormalities. The T2-weighted and fat-suppressed MRI scans revealed a mass with high signal intensity just posterior to the patellar tendon (Figure 1). Her laboratory tests, including coagulation parameters, were all within normal values.

The radiologists included synovial sarcoma in the differential diagnosis, and we performed an excisional biopsy of the mass through the anterior longitudinal incision. We decided to perform open surgery because of the unknown characteristics of the lesion. The lesion, which measured 3cm × 2cm, was excised fully.

**Figure 2:**
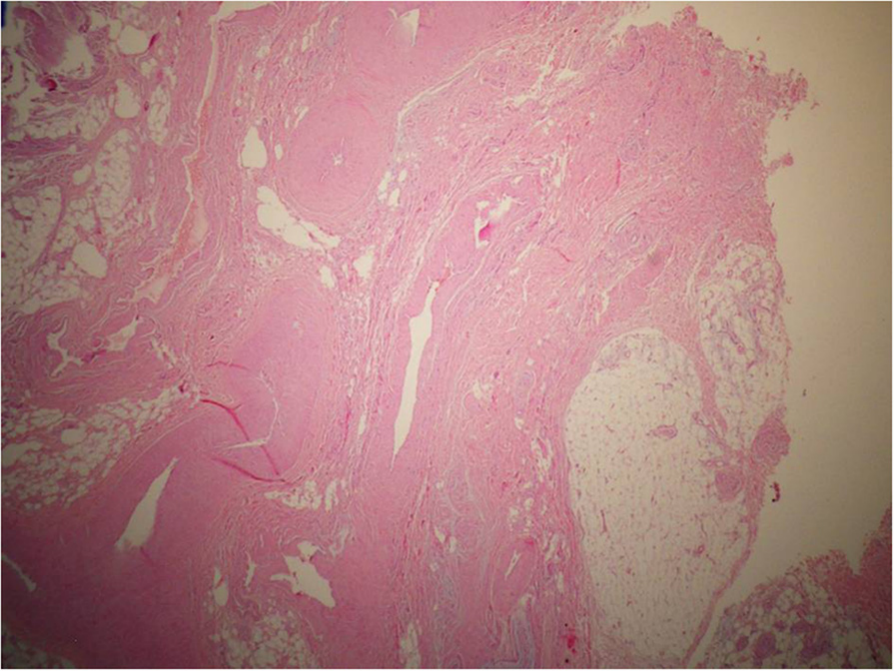
Arterial and venous vascular structures between fat, connective and peripheral nerve sections (hematoxylin and eosin stain; ×50 original magnification).

**Figure 3:**
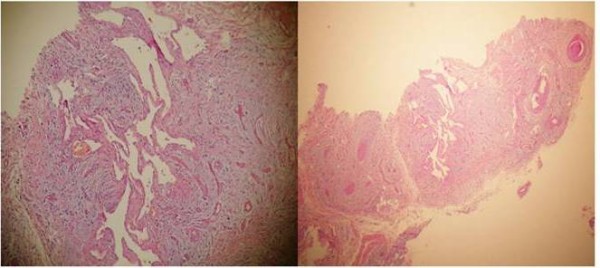
(**a) and (b) Vascular regions that form clefts in a canalicular pattern (hematoxylin and eosin stain; ×200 and × 50 original magnification, respectively).**

The excised material was soft and encapsulated macroscopically. Microscopically, arterial and venous vascular structures were found to be spread among the fat, connective and peripheral nerve tissues (Figure 2,3). The patient was well 6 months after the operation, with no pain and swelling at her knee. She also had full range of knee motion.

## Discussion

Synovial hemangiomas and hemangiohamartomas are two entities that are both rare and similar in nature. Hemangiohamartomas involve the synovia with the fat and connective tissue. In addition to pain, swelling and palpable mass, they may cause hemarthrosis [9] and were first described by Eugène Bouchut in 1856 [3,10].

The differential diagnosis for hemangiohamartoma should include pigmented villonodular synovitis, synovial sarcoma, rheumatoid arthritis, juvenile chronic and arthritis, hemophilic arthropathy and lipoma arborescens.

Arthroscopic excision in cases of focal or pediculated and appropriate-size lesions may be an alternative to open surgical resection. In cases of pure synovial hemangioma, selective embolization of feeder vessels might also be an alternative to surgery [11-13].

Plain radiographs are usually of poor diagnostic value. In less than 5% of patients, periosteal reaction, cortical destruction, discrepancy in leg length or even arthropathy simulating hemophilia may be seen [12,13]. MRI is the most useful diagnostic tool, especially on T2-weighted images, on which the lesion exhibits a high signal due to blood in vascular spaces [5].

## Conclusion

Although hemangiohamartomas are not true neoplasms, they may cause knee pain, swelling and hemarthrosis that warrant surgical resection. This lesion, although rare, should be considered as part of a differential diagnosis, especially in teenagers and young adults.

## Consent

Written informed consent was obtained from the patient for publication of this manuscript and any accompanying images. A copy of the written consent is available for review by the Editor-in-Chief of this journal.

## Competing interests

The authors declare that they have no competing interests.

## Authors’ contribution

SS, MSS and EU contributed to the conception and design of this case report and carried out the literature research, manuscript preparation and manuscript review. HC was involved with the case and the writing of the manuscript as well as the general management of the patient. KO revised the manuscript for important intellectual content. All authors read and approved the final manuscript.
